# Y Chromosome, Hypertension and Cardiovascular Disease: Is Inflammation the Answer?

**DOI:** 10.3390/ijms20122892

**Published:** 2019-06-13

**Authors:** Shanzana I. Khan, Karen L. Andrews, Garry L. Jennings, Amanda K. Sampson, Jaye P. F. Chin-Dusting

**Affiliations:** 1 Department of Pharmacology, Monash University, Clayton, Victoria 3800, Australia; karen@vividhomesolutions.com.au (K.L.A.); jaye.chin-dusting@baker.edu.au (J.P.F.C.-D.); 2 Baker IDI Heart and Diabetes Institute, Melbourne, Victoria 3004, Australia; garry.jennings@baker.edu.au (G.L.J.); amanda.sampson@monash.edu (A.K.S.)

**Keywords:** vascular dysfunction, sex differences, immune-mediated hypertension

## Abstract

It is now becomingly increasingly evident that the functions of the mammalian Y chromosome are not circumscribed to the induction of male sex. While animal studies have shown variations in the Y are strongly accountable for blood pressure (BP), this is yet to be confirmed in humans. We have recently shown modulation of adaptive immunity to be a significant mechanism underpinning Y-chromosome-dependent differences in BP in consomic strains. This is paralleled by studies in man showing Y chromosome haplogroup is a significant predictor for coronary artery disease through influencing pathways of immunity. Furthermore, recent studies in mice and humans have shown that Y chromosome lineage determines susceptibility to autoimmune disease. Here we review the evidence in animals and humans that Y chromosome lineage influences hypertension and cardiovascular disease risk, with a novel focus on pathways of immunity as a significant pathway involved.

## 1. Introduction

The male-specific region of the Y chromosome (MSY), which constitutes 95% of its length, does not undergo recombination and is thus inherited virtually intact from father to son [1]. Encompassing merely 27 protein-coding genes, it is one of the smallest chromosomes in the human genome [1]. The essential role of the Y chromosome in male sex determination has overshadowed the possibility that it may exert pleiotropic effects, despite early observations that the main sex determining gene *sry* is expressed in a number of tissues central to cardiovascular regulation such as the human kidney, the adrenal gland and the brain [2]. Turner et al. were the first to comment that such an expression profile in such a vast array of organs unrelated to testis determination across so many species is not consistent with the functions of the gene being restricted to the induction of male sex [2]. Importantly, the DNA binding domain of *sry* is highly conserved between species [3]. However, up until recently, the haploid nature of the Y chromosome has caused its routine exclusion in genome-wide association studies. Once dubbed ‘a genetic wasteland’, the Human Genome Project confirmed the paucity of genes and high degree of repetition in the Y. However, consistent with findings in animal consomic models by Turner et al., it was found that half the active genes are expressed in nongonadal tissues. 

A large paradigm shift has occurred in recent years regarding the biological importance of the Y in human disease. The initial impetus to study the role of the Y in cardiovascular disease emanated from well-established sexual dimorphism in hypertension and coronary events [4,5,6,7,8,9,10]. Since then, accumulating evidence in animals and humans suggests Y chromosome lineage may be one of the strongest genetic determinants of coronary artery disease to date [11]. Unexpectedly, it was found that elevated coronary artery disease risk associated with Y chromosome lineage occurred independently of traditional risk factors but was associated with pathways of immunity. Furthermore, recent studies in mouse models have shown that Y chromosome lineage is a significant determinant of autoimmunity [12]. As it has become increasingly apparent that T cells play a role in hypertension and disease sequelae, we propose that Y chromosome modulation of adaptive immunity may be a hitherto-unexplored mechanism underlying men’s susceptibility to hypertension and cardiovascular disease. Here we review the evidence from man and rodent supporting a role for the Y in hypertension and cardiovascular disease with a new focus on inflammation and immunity as a significant pathway involved.

## 2. Y Chromosome and Hypertension: Studies in Rodents

Animal models enable the dissection of complex, multifactorial phenotypes through the control of environmental conditions and isolation of genes. Such studies have made significant contributions to our understanding of the role of sex hormones in blood pressure (BP) regulation and cardiovascular disease, which is extensive reviewed elsewhere [13]. Animal studies delineating associations between the Y and cardiovascular phenotypes are summarized in Table 1. Two main animal models have been utilised to isolate the contribution of the Y chromosome and autosomes to BP. The four core genotype (FCG) mouse model was created to investigate the role of sex chromosome complement independent of sex hormones [14]. The deletion of the *sry* gene from the Y chromosome results in a mouse possessing the XY complement that does not develop testes or produce testosterone. Conversely, the insertion of *sry* onto an autosome in the female results in a female possessing male sexual characteristics but possessing the XX chromosome complement. This enables a 2 by 2 comparison (XYM, XYF, XXM, XXF) which distinguishes the effects of sex chromosome complement independent of sex hormones. Using this model, Ji et al. showed that the presence of the Y chromosome is associated with a blunted pressor response to angiotensin II [15]. This is a surprising finding given a blunted pressor response is consistently observed in female mice in response to angiotensin II compared with males [16,17,18] and highlights that the effects of sex chromosome complement may not align with sex hormones. However, this model has a number of limitations. As we have previously highlighted [19], this approach takes for granted that genes on the Y chromosome will exert the same effects in the absence of typical male prompts, such as the presence of testosterone. Furthermore, the exploration of multiple functions of *sry*, in addition to its sex-determining properties, is precluded.

In contrast to the FCG model, consomic models assess the contribution of Y chromosome lineage to BP. Consomic models created in the stroke-prone spontaneously hypertensive rat (SHRSP) and its close relative, the SHR, have delineated a significant role for Y chromosome lineage in BP regulation [21,23]. We and others have shown that introgression of the SHRSP Y chromosome into the WKY background results in a 12–25 mm Hg increase in BP and vice versa [21,26], which represents up to a third of the BP difference between SHRSP and WKY and is the largest contribution to BP from any of the SHRSP chromosomes. A number of investigations have been conducted to elucidate the mechanisms underlying this effect. 

SHRSP strain with the normotensive WKY Y chromosome (referred to as SP.WKY_Gla_Y) shows improved salt-sensitivity compared with the SHRSP strain with its native hypertensive SHRSP Y chromosome [22]. Coronary collagen deposition is also lower in the SP.WKY_Gla_Y and WKY compared with the SHRSP [23], while renal norepinephrine turnover is higher in the WKY.SP_Gla_Y and SHRSP compared with the WKY [23]. While no Y-chromosome-dependent differences in plasma testosterone levels are seen in this model, it has been suggested that an earlier rise in testosterone induced by introgression of the SHRSP Y chromosome into the WKY background may be responsible for increased norepinephrine sensitivity, given testosterone has been shown to increase α1 adrenergic receptor density [24]. 

Significant interactions have been revealed between the Y chromosome and the renin angiotensin system (RAS). While only one isoform exists in humans, multiple isoforms of *sry* exist in rats and renal overexpression of *sry3*, a candidate gene located exclusively on the SHRSP Y chromosome, mediates a 50% increase in renal sodium reabsorption and glomerular filtration [37]. This was associated with upregulated angiotensinogen, renin and ACE promoter activity and downregulation of ACE2 promoter activity [25], thereby enhancing the vasoconstrictor RAS and dampening the vasodilatory RAS. Furthermore, renal *sry3* overexpression increases renal angiotensin II levels and BP in vivo [37]. We have also shown that plasma angiotensin II levels are Y-chromosome-dependent in this model [26] and that Y chromosome lineage modulates the intrarenal responsiveness to angiotensin I and angiotensin (1–7), as well as the ratio of vasodilatory to vasoconstrictive RAS receptors [26].

The sympathetic nervous system (SNS) has also been shown to contribute to Y-chromosome-dependent regulation of BP. Adrenal gland chromagranin A and tyrosine hydroxylase levels are higher in the WKY.SP_Gla_Y compared to the WKY [27], while sympathectomy abolishes BP differences between the WKY and WKY.SP_Gla_Y [28]. Furthermore, *sry1*, another isoform of the testes-determining gene *sry* in rat, enhances tyrosine hydroxylase activity [38,39]. The latter effects were shown to be mediated via interactions at the AP1 site, which is also ubiquitous in the human adrenal gland [40]. Furthermore, Y-chromosome-dependent differences in salt sensitivity have been shown to be SNS-dependent [23] and renal sry1 electroporation increases markers of SNS activity [39]. 

Interestingly, both Y chromosome lineage and sex chromosome complement have been shown to influence adiposity and lipid metabolism. Studies in gonadectomised FCG mice revealed that the XX chromosome complement accounted for greater weight gain in response to high fat diet (HFD) feeding due to greater accumulation of adipose tissue, accompanied by metabolic disturbances such as fatty liver, elevated total blood cholesterol levels and elevated insulin levels [29]. This was found to be mediated, at least in part, by increased food intake in the light phase of the circadian cycle [30]. The presence of two X chromosomes was also shown to increase growth hormone mRNA in the preoptic area of the hypothalamus [31]. These findings are striking, given they are at complete odds with well-described resistance to weight gain observed in female mice fed a high fat diet, attributable to oestrogen [41]. Conversely, increased X chromosome gene dosage has been shown to elevate high density lipoprotein (HDL) levels [32], which is associated with cardioprotection due to the ability of HDL to scavenge and eliminate triglycerides, indicating characterization of sex chromosome complement and metabolic profile is not so simplistic. Variations in the Y have been shown to influence lipoprotein profiles in both rodent [33] and man [42]. Given the intimate association between obesity and hypertension, it is important to note that this has also been demonstrated in SHRSP, with assessment of consomic strains showing that replacement of the SHRSP Y chromosome with that of its normotensive counterpart (SP.WKY_Gla_Y) lowered triglyceride levels and improved glucose tolerance, mediated at least in part via an interaction with chromosome 2 [34].

## 3. Y Chromosome, Cardiovascular Disease and Hypertension: Human Studies

Mapping and sequencing of the Y chromosome has been enabled with the recent advent of genetic technologies. However, interest in Y-linked traits has been present since the second half of the twentieth century. One of the first studies to suggest that BP could be inherited through father to son transmission showed that male students born to hypertensive fathers had higher BP than female students born to hypertensive fathers, although hypertensive fathers did not have higher BP than male students born to hypertensive mothers, which did not support Y-inheritance [43]. However, this work prompted further investigations into Y-linked BP inheritance and was succeeded by population studies indicating that BP status in men is determined by paternal rather than maternal BP status.

Further impetus to investigate the Y and BP was derived from studies that correlated variations in the Y chromosome with BP. Before the introduction of phylogenetic tree classifications that enable Y chromosome lineages to be classified according to single nucleotide polymorphisms, only a few known variants of the Y chromosome were known. The HindIII restriction site divides Y chromosome into 2 classes. HindIII variation was shown to account for 1.44 to 6.2 mmHg of BP in three separate studies [44,45,46], although this was not the case for others.

The identification of biallelic single nucleotide polymorphisms enables the classification of MSY into haplogroups. This significant advance has enabled the study of associations between Y and complex diseases and has potential to fulfil significant gaps in knowledge left by genome-wide association studies (GWASs) that routinely excluded the Y from analysis. Using this approach, twenty major haplogroups have been identified in European men. It has been shown that one of the most common haplogroups of the Y chromosome, haplogroup I, is associated with a 50% increase in the risk of coronary artery disease compared with other haplogroups [47]. This renders Y chromosome lineage as the single most significant genetic determinant of cardiovascular disease to date. 

Associations between Y haplogroup and other cardiovascular diseases have also been delineated. Importantly, these include investigations conducted in cohorts of non-European males, enabling analysis of a more diverse set of haplotypes. Haplogroup K was associated with a 50% increase in the risk of atherosclerotic plaque and femoral artery bifurcations compared to other haplogroups in Cypriot men [48]. However, investigation of histological vessel wall parameters including lipid, collagen and smooth muscle content in Dutch men who had undergone carotid endarterectomy or open aneurysmal repair did not reveal any differences between Y haplogroups [49]. 

To date, there is a paucity of studies investigating the association between Y haplogroup and BP in large, diverse cohorts of men. Only one study has assessed the influence of polymorphisms in the Y on cardiovascular parameters in Asian men and found that Y chromosome Alu insertion polymorphism (YAP) did not influence cardiovascular risk factors in Japanese men, although conclusions were significantly limited by low power due to low sample size [50]. Although the association between haplogroup I and coronary artery disease was found to be independent of BP [11,47], the association between other common haplogroups and BP has not been investigated. Furthermore, higher systolic BP was suggested to be the potential mediator of elevated risk of atherosclerosis and femoral artery bifurcations associated with haplogroup K [48]. The availability of extensive databases such as the UK Biobank that holds phenotypic data of 230,000 men and phylogenetic tree [51] would greatly facilitate investigations into whether Y-chromosome-dependent variations in BP in rat are paralleled in man.

## 4. Y Chromosome and Vascular Function

Vascular dysfunction is a hallmark of hypertension and an important precursor to cardiovascular events [52,53,54,55,56]. It is typified by an impaired ability of endothelial cells to induce underlying smooth muscle relaxation, resulting in vessel stiffness and reduced perfusion of key organs. Both the RAS and the SNS are significant modulators of vascular function, with the net effect of activation of both these systems being to increase vascular tone. Given it had previously been shown that *sry3* found exclusively on the SHRSP Y chromosome is a significant regulator of the RAS in vitro [25], we decided to investigate the in vivo effects of intrarenal infusion of RAS peptides. Surprisingly, we found a dampened constrictor response to angiotensin II in intrarenal arteries in the SHRSP that was not Y-chromosome-dependent, as similar responses were observed in SHRSP and SP.WKY_Gla_Y vs WKY and WKY.SP_Gla_Y [26]. However, we observed that the constrictor response to angiotensin I and angiotensin 1–7 was blunted in SHRSP compared to that to WKY, which was reversed in SP.WKY_Gla_Y. This was accompanied by greater plasma levels of angiotensin 1–7 and greater renal mRNA expression of angiotensin type 2 and Mas Receptors in SHRSP compared to WKY, which was reversed in SP.WKY_Gla_Y. However, in this study, we were unable to investigate protein levels of these receptors, which would be expected to be downregulated in response to higher plasma angiotensin (1–7) levels. This remains to be determined. Our findings contrast with previous in vitro observations of upregulated ACE promoter activity and downregulated ACE2 promoter activity mediated by *sry3* on the SHRSP Y chromosome [25]. As the promoter of ACE contains two sheer response elements [57], it is unsurprising that the in vivo consequences of the SHRSP Y chromosome do not align with these previous findings in vitro.

These findings of Y-chromosome-dependent differences in intrarenal vascular responsiveness to the RAS prompted further investigations from our group into Y-chromosome-dependent differences in vascular function. We found impaired vascular responses to the endothelium-dependent vasodilator acetylcholine were impaired in the SHRSP aorta compared to WKY, which was reversed in the SP.WKY_Gla_Y, although no differences were observed between WKY and WKY.SPG_la_Y [35]. Interestingly, nitric oxide levels were unaffected in SHRSP compared to WKY, with vascular dysfunction attributable to elevated production of constrictor prostanoids, which was abolished in SP.WKY_Gla_Y. These effects were also seen in the renal and intrarenal arteries. As we found markedly augmented prostacyclin production in the SHRSP aorta compared to the other three strains, which was paralleled by prostacyclin (IP) receptor dysfunction, we suggest that the identity of the constrictor prostanoid is prostacyclin acting as a paradoxical vasoconstrictor. As inhibition of the vasoconstrictive thromboxane receptor (TP) reversed vascular dysfunction in SHRSP, we suggest that this may be the mediator of prostacyclin’s vasoconstrictive effects, in line with observations by others in SHR [58]. Given vascular dysfunction is one of the strongest predictors for atherosclerosis and thrombotic events, these findings that the Y influences vascular cyclo-oxygenase (COX) activity could have significant implications for the treatment of cardiovascular disease in males.

## 5. Y Chromosome and Inflammation

Strikingly, investigations of Y haplogroup and association between coronary artery disease revealed the unexpected finding that haplogroup I appeared to mediate male coronary artery disease risk independent of any traditional risk factors, including dyslipidaemia, high BMI, alcohol consumption or smoking [11,47]. Conversely, transcriptome wide analysis highlighted that of 30 Kyoto Encyclopedia of Genes and Genomes (KEGG) pathways differentially regulated by haplogroup I and carriers of other haplogroups, nineteen were pathways of autoimmunity and adaptive immunity. Inflammation is the most significant driver of atherosclerosis and vascular disease with augmented macrophage infiltration into the vascular wall being a significant player, prompting the authors to examine differences in macrophage expression of genes of MSY between haplogroup I and other haplogroups. Of 14 X-degenerate genes of MSY expressed in macrophages, UTY and PRKY were found to be downregulated in men of haplogroup I lineage. PRKY is thought to encode a protein kinase with significant signalling functions [59], and UTY and its X paralogue UTX are associated with inflammatory responses of macrophages, including cytokine production [60], and encode a minor histocompatibility antigen that has a significant role in male stem cell allograft rejection [61]. These findings are intriguing given emerging evidence that macrophage phenotypes traditionally classed as anti-inflammatory may play a significant role in plaque formation and coronary diseases [62].

Further evidence for the role of Y chromosome lineage being a significant determinant of inflammation comes from studies in man and rodent in settings of immunodeficiency and autoimmune disease. Investigations into a cohort of HIV positive patients of European American descent revealed men of halogroup I lineage exhibited faster progression of the condition to AIDS and a higher mortality rate at 7 years post-infection [63]. These effects were attributable to dysregulated immunity in haplogroup I men compared to other haplogroups, as lower CD4+ T cell counts and delayed responses to viral load suppression in response to antiretroviral therapy were observed. Higher rates of malignancy observed in haplogroup I men was also consistent with impaired immune responses in these men compared with other haplogroups.

Y chromosome lineage was also a significant determinant of susceptibility to experimental allergic encephalomyelitis (EAE) and myocarditis in consomic strains of mice [12]. Differential copy numbers of mouse Y genes SLY and RBMY were suggested to account for Y-chromosome-dependent differences in disease severity. Three hundred and ninety-eight differentially expressed Y chromosome transcripts were identified in macrophages in strains of rat that were more prone to disease than others. Intriguingly, CD4+ T cells from men with the human correlate of EAE, clinically isolated syndrome (CIS), also showed differential expression of the same MSY genes that were identified in mouse studies. Altogether, there is strong evidence of a locus on the Y modulating autoimmunity, with common pathways possibly shared between rodent and man.

## 6. Y Chromosome, T Cells and Hypertension

Hypertension is the leading risk factor for cardiovascular events, with studies consistently observing elevated BP in males compared with females mirroring cardiovascular mortality rates that are triple those of females. An immune component to hypertension is now well established, with increased T cell infiltration into target cardiovascular organs contributing to end-organ damage through the production of ROS and inflammatory cytokines [64,65,66,67,68,69,70,71,72]. There is also evidence that adaptive-immune regulation of blood pressure is sex-specific. Males SHRs exhibit a more pro-inflammatory renal T cell profile than females [73]. Furthermore, the mechanisms regulating immune cell profiles in target cardiovascular organs such as the kidney are sexually dimorphic, with males being less sensitive to the pro-inflammatory effects of NO inhibition, the bioavailability of which is lower in the male versus female SHR kidney [74]. The most compelling evidence depicting sex-specific regulation of blood pressure by the adaptive immune system comes from adoptive transfer studies. Peak mean arterial pressure, renal T cell infiltration and splenic T cells producing pro-inflammatory cytokines TNFα and IL-17 were higher after adoptive transfer of male versus female T cells into male *RAG-1^-/-^* mice, and was accompanied by lower renal mRNA expression of interleukin-10 [75]. Conversely, adoptive transfer of male T cells into male RAG-1^-/-^ hosts results in significantly elevated BP, renal and subfornical T cell infiltration compared to female hosts, suggesting sex-specific interactions between T cells and host milieu [76]. Furthermore, adoptive transfer of male T cells into male *RAG-1^-/-^* mice was associated with increased perivascular T cell infiltration than if the T cells were derived from female wild-type mice [75]. Importantly, in vitro treatment with sex hormones does not influence T cell cytokine profiles [77], and castration and ovariectomy both elicit pro-inflammatory effects [78], suggesting sex hormones cannot entirely account for sex-specific regulation of BP by the adaptive immune system.

Given well-established sex differences in adaptive immunity and mounting evidence that Y chromosome haplogroup influences immunity to in turn mediate susceptibility to cardiovascular disease, we investigated whether Y-chromosome-dependent differences in BP in rodent were associated with Y-chromosome-dependent differences in T cell infiltration [36]. We have previously shown Y-chromosome-dependent differences in BP in consomic strains, with introgression of the SHRSP Y chromosome into the WKY background increasing BP and vice versa [26]. Consistent with augmented adaptive immunity in hypertension, T cell infiltration was higher in SHRSP aortae and kidney compared with WKY, which was abolished with replacement of the SHRSP Y chromosome, although introgression of the SHRSP Y chromosome into WKY reduced T cell infiltration into kidneys only. Intriguingly, adoptive transfer of SHRSP but not SP.WKY_Gla_Y T cells into the latter resulted in systolic BP increase and vascular dysfunction. We also investigated the mechanisms of vascular dysfunction associated with augmented perivascular T cell infiltration and found T cells may directly mediate vascular dysfunction through stimulation of cyclo-oxygenase (COX) in the vascular wall. Given we had previously shown Y-chromosome-dependent differences in vascular function with differential regulation of COX activity as an intermediate phenotype governing this effect [35], it appears that differential T cell infiltration may also play a role in this Y chromosome-mediated phenotype. Importantly, elevated production of reactive oxygen species from T cells stimulating COX in the vascular wall was found to be a significant mechanism underpinning T cell-mediated vascular dysfunction. Although we did not distinguish which isoform of COX was at play, we postulate that it was the COX-1 isoform, given evidence this is the isoform most implicated in vascular dysfunction in SHRSP [79]. It remains to be investigated whether other well-described cardiovascular phenotypes ascribed to the SHRSP Y chromosome interact with adaptive immunity. Furthermore, although unlikely, bone marrow transfer studies in these consomic strains would further confirm the differences in T cell infiltration are not secondary to BP differences. Importantly, MSY genes mediating these effects are yet to be isolated. Thus, future transcriptomic studies in T cells isolated from these consomic strains will be critical in determining if similar mechanisms mediate male susceptibility to hypertension.

## 7. Conclusions

The perception of the Y chromosome as a defunct piece of genetic machinery apart from its sex-determining functions has undergone significant revisions in the last couple of years. While consomic models have provided significant insights into Y chromosome regulation of traditional cardiovascular pathways, human studies suggest that Y chromosome lineage influences cardiovascular risk through modulation of adaptive immunity. We have provided compelling evidence that Y chromosome lineage predicts T cell infiltration in key cardiovascular organs to in turn influence BP. It remains to be determined whether these studies are mirrored in man. We suggest that an analysis of extremes approach be taken to GWAS data sets to determine the incidence of Y haplogroups in hypertensive vs. normotensive males. If any haplogroups are found to occur more frequently in hypertensive compared with normotensive males, transcriptomic analysis of T cells highlighting differential gene expression may provide significant insights into pathways of immunity differentially regulated by Y chromosome lineage. Given the significant burden of hypertension and cardiovascular disease, which are significantly higher in males compared to females, such initiatives could result in significant public health benefits. Future studies examining whether Y chromosome lineage influences therapeutic efficacy of established antihypertensive and anti-inflammatory agents would also be of great value, possibly even paving the way for using a Y chromosome phylogenetic tree in prescribing choices.

## Figures and Tables

**Table 1:** Summary of rodent studies on Y chromosome lineage and cardiovascular phenotypes.

Reference	Study Conducted In	Major Results
Ji et al., 2010 [15]	FCG mouse model	Presence of Y chromosome is associated with blunted pressor response to angiotensin II
Ely and Turner, 1990 [20]	SHR consomic strains	First study to show that there is a significant BP locus on the SHR Y chromosome.
Davidson et al., 1995 [21]	SHRSP consomic strains	First study to show significant BP locus on the SHRSP Y chromosome
Negrin et al., 2001 [22]	SHRSP consomic strains	Y chromosome lineage influences salt sensitivity
Ely et al., 2000 [23]	SHR consomic strains	Y chromosome lineage influences coronary collagen deposition
Ely et al., 2000 [23]	SHR consomic strains	Y chromosome influences renal norepinephrine turnover
Ely et al., 1995 [24]	SHR consomic strains	SHR Y chromosome mediates an early testosterone rise
Ely et al., 2011 [25]	SHR consomic strains	*Sry3* upregulates angiotensinogen, renin and ACE promoter activity in vitro and mediates an increase in sodium reabsorption
Sampson et al., 2014 [26]	SHRSP consomic strains	Origin of Y chromosome influences intrarenal vascular responsiveness to RAS peptides
Ely et al., 1997 [27]	SHR consomic strains	Y chromosome lineage influences indexes of SNS activity
Wiley et al., 1999 [28]	SHRSP consomic strains	Sympathectomy abolishes BP differences between WKY and WKY.SP_Gla_Y
Chen et al., 2012 [29]	FCG mouse model	XX chromosome complement mediates increased adiposity and metabolic disturbances in mice fed HFD
Chen et al., 2015 [30]	FCG mouse model	XX chromosome complement accounts for increased food intake in mice on HFD
Bonthuis et al., 2013 [31]	FCG mouse model	XX chromosome complement increases growth hormone gene in preoptic area of hypothalamus
Link et al., 2015 [32]	FCG mouse model	XX chromosome complement accounts for elevated HDL levels
Suto and Satou et al., 2019 [33]	Y consomic mouse strains	Y chromosome lineage accounts for differences in lipoprotein profiles
Strahorn et al., 2005 [34]	SHRSP consomic strains	Y chromosome lineage determines metabolic phenotypes, mediated in part via an interaction with chromosome 2
Khan et al., 2018 [35]	SHRSP consomic strains	Y chromosome lineage accounts for vascular dysfunction in the SHRSP through influencing cyclo-oxygenase activity
Khan et al., 2019 [36]	SHRSP consomic strains	Y chromosome lineage determines perivascular and renal T cell infiltration to in turn influence vascular function
